# Pott’s Puffy Tumour – Rare and Forgotten, yet Relevant and Life-Threatening: Case Report

**DOI:** 10.15388/Amed.2025.32.1.6

**Published:** 2025-02-18

**Authors:** Himraj Phukan, Donboklang Lynser, Gareth Yobel Lyngwa, Chhunthang Daniala, Himesh Barman, Tamajyoti Ghosh

**Affiliations:** 1 North Eastern Indira Gandhi Regional Institute of Health and Medical Sciences, Shillong, India

**Keywords:** craniotomy, MRSA, osteomyelitis, Pott’s puffy tumour, subdural empyemas, kraniotomija, MRSA, osteomielitas, Potto pūlingas auglys, subdurinė empiema

## Abstract

Pott’s Puffy Tumour (PPT) is an inflammation and swelling of the forehead secondary to osteomyelitis of the frontal bone. Multiple aetiologies are associated with this condition, predominately involving a prior sinus surgery or a direct trauma to the frontal bone. There can be intracranial extension with an epidural, subdural as well as intracerebral abscess. We present the case of an 8-year-old boy who presented with swelling in his forehead secondary to trauma. His general condition was poor, and further evaluation revealed multiple subdural empyemas. Although PPT is rare in this modern era of antibiotics, it should be kept as a differential for any inflammation of the forehead. Timely diagnosis and the appropriate treatment by a multidisciplinary team are indispensable in reducing the morbidity and mortality associated with this case.

## Abstract (it)

Potto pūlingas auglys (angl. *Pott‘s Puffy Tumour* (PPT)) – antrinis kaktos uždegimas ir patinimas, atsiradęs dėl priekinio kaulo osteomielito. Ši būklė siejama su įvairiomis etiologijomis, dažniausiai susijusi su ankstesne sinuso operacija arba tiesiogine priekinio kaulo trauma. Gali būti intrakranijinis išplitimas su epiduriniu, subduriniu ir intracerebriniu abscesu. Pateikiame 8 metų berniuko, kuriam antrinė trauma sukėlė kaktos patinimą, atvejį. Jo bendra būklė buvo prasta, o atlikus tolesnį vertinimą nustatyta daugybinė subdurinė empiema. Nors PPT šiais moderniais antibiotikų laikais pasitaiko retai, jis turėtų būti laikomas diferencine bet kokio kaktos uždegimo priežastimi. Laiku nustatyta diagnozė ir tinkamas daugiadalykinės komandos atliktas gydymas yra būtini siekiant sumažinti su šiuo atveju susijusį sergamumą ir mirtingumą.

## Introduction

*Pott’s Puffy Tumour* (PPT) refers to osteomyelitis of the frontal bone accompanied by a subperiosteal abscess. Sir Percival Pott, a surgeon from London first described this condition in 1768 in the context of a forehead trauma and later, in 1775, in relation to an early sinusitis [1]. It has become rare in modern times due to the advent of broad-spectrum antibiotics; however, it can be dangerous, thereby requiring proper clinical suspicion and diagnosis. We present a case of PPT in an adolescent child with intracranial extension.

## Case presentation

**Figure 1:**
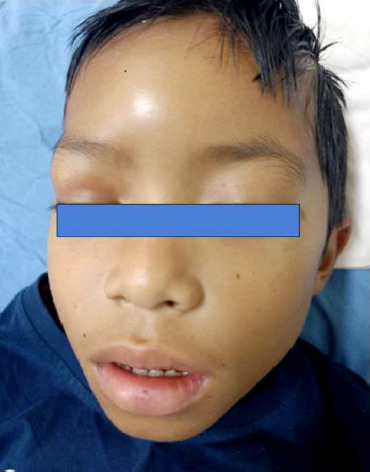
A case of Pott’s puffy tumour showing a boggy swelling over the right forehead extending to the right upper eyelid

An 8-year-old boy came to our hospital referred from a primary health care center with symptoms of fever and cough for 7 days, abnormal jerky movements of the body with an altered sensorium over the last 3 days. His natal and immunization history was remarkable. There was no history of similar episodes in the past. The patient had a history of trauma to his forehead while playing around 10 days back, and the patient presented with a reddish swelling at the site of the injury (see Figure 1).

He was allegedly hit by his brother with a wooden bat while playing. The patient’s GCS was poor, and thereby he was directly admitted to the *Paediatric Intensive Care Unit* (PICU) followed by urgent intubation. A working diagnosis of Meningoencephalitis was made, and radiological investigations were undertaken. The hemoglobin of the patient was low (8.8 gm%), with a WBC count of 37 x 10^3^/mm^3^. ESR (85 mm at the end of 1 hour) and CRP (131 mg/L) were also raised. His deep tendon reflexes were absent with a hypotonic tone.

**Figure 2:**
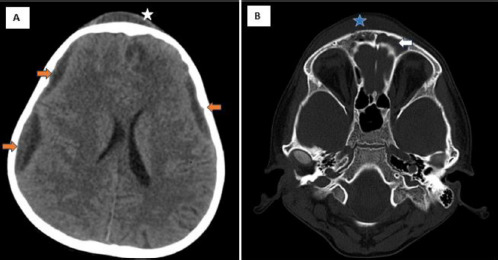
NCCT Images of an 8-year-old boy with Pott’s puffy tumour showing: **A** – Axial image in the parenchymal window showing subdural hypodensities along bilateral cerebral hemispheres (orange arrows) with a right frontal extra-calvarial hypodensity (white star); **B** – Axial image in the bone window showing mucosal thickening in bilateral frontal sinuses (white arrow) with erosions in the right frontal bone including the anterior wall of the frontal sinus (blue star)

NCCT brain revealed mucosal thickening in bilateral frontal sinus with bilateral subdural hypodensities. Multiple small erosions were noted in right frontal bone including the anterior wall of the frontal sinus (Figure 2).

**Figure 3:**
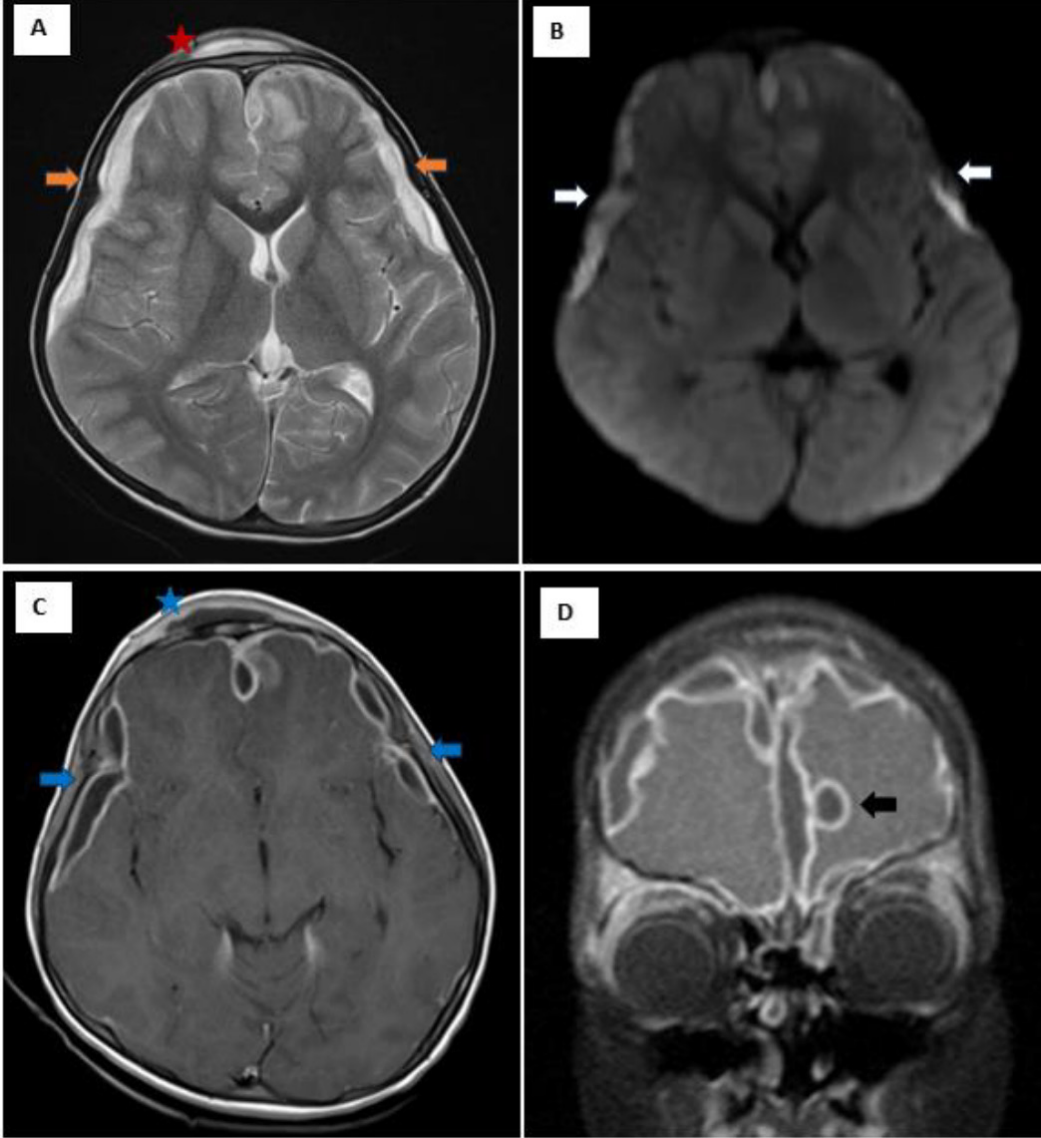
MRI Images of an 8-year-old boy with Pott’s puffy tumour showing: **A** – Axial T2 images showing subdural collections (orange arrows) and a subperiosteal abscess (red star) overlying the frontal bone predominately on the right side; **B** – DWI images showing diffusion restriction of the collections (white arrows); **C** – Axial contrast-enhanced T1 sequence showing peripherally enhancing subdural collections (blue arrows) and sub-periosteal abscess (blue star); **D** – Coronal contrast-enhanced T1 sequence showing extensive peripherally enhancing subdural collections with a parafalcine collection causing indentation of the left frontal lobe (black arrow)

The patient was later taken up for a contrast enhanced MRI, which showed that multiple subdural empyemas are observed along the bilateral cerebral convexities and falx. There was also a peripherally enhancing sub-galeal collection noted overlying the affected frontal bone and the frontal sinus predominantly on the right side, which was seen tracking to the right pre-septal soft tissues (see Figure 3).

A diagnosis of Pott’s puffy tumour secondary to frontal sinusitis was made, and neurological opinion was sought. The CSF study was unremarkable. Bi-coronal flap with bifrontal craniotomy and drainage of the subdural empyema was performed. Further evaluation for his anaemia profile revealed an HbE trait. Pus sent for culture and gram staining detected *Methicillin-Resistant Staphylococcus Aureus* (MRSA). The patient was assigned a six-week administration of Vancomycin with an uneventful post-operative course. The patient improved and was discharged on the follow-up.

**Figure 4:**
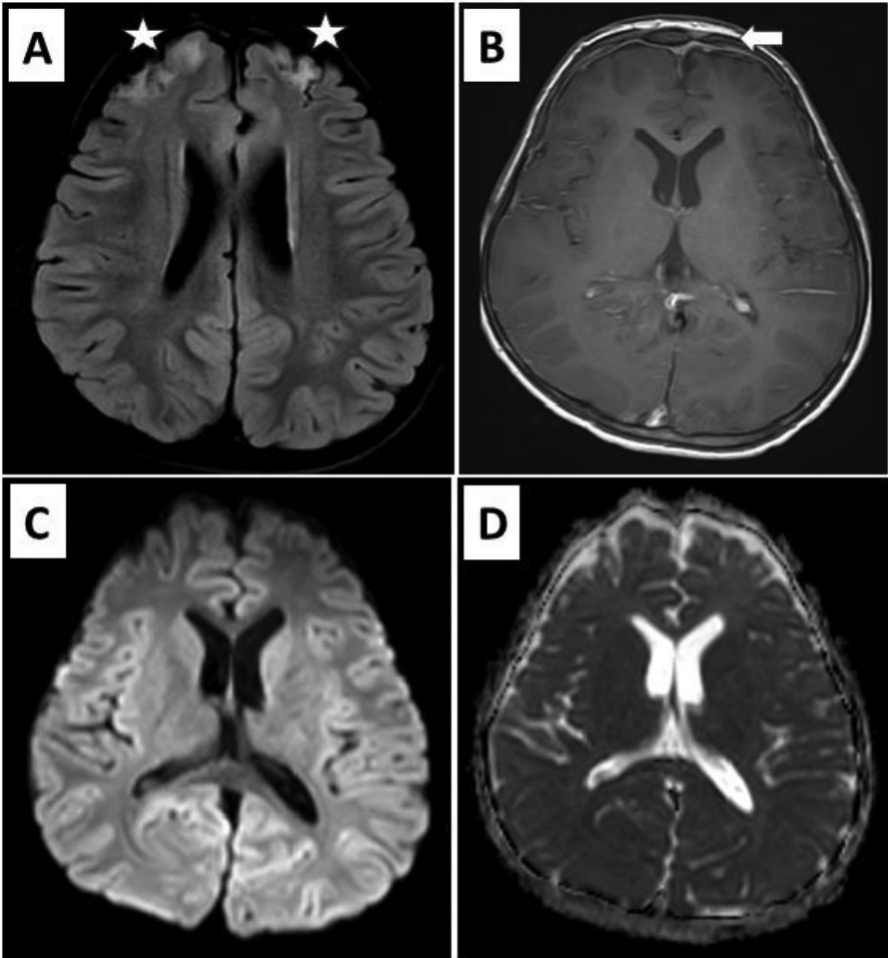
Follow-up MRI images taken after 8 months of an 8-year-old boy with Pott’s puffy tumour showing: **A** – Axial T2 FLAIR image showing bifrontal cortical foci of increased signal intensities (white stars) suggestive of gliotic changes; **B** – Axial contrast-enhanced images showing associated smooth pachymeningeal thickening (white arrow); **C** and **D** – DWI and ADC images, respectively, showing no features of residual collections

A follow-up MRI was performed after 8 months, which revealed complete resolution of the subdural collections. However, mild bifrontal gliotic changes were seen with smooth pachymeningeal thickening, suggestive of sequelae changes (Figure 4). The patient is doing fine clinically, and an adequate recovery has been achieved according to the parents. He is attending school regularly and resuming his normal playful life.

## Discussion

PPT is an inflamed swelling of the forehead indicating an underlying osteitis. Frontal sinuses are usually pneumatized by 2 years of age, and they attain the size of adulthood by adolescence. PPT is a very serious complication of frontal sinusitis, and, if left untreated, it can cause vascular compromise and secondary thrombophlebitis. This, in turn, may lead to bone necrosis and pose a risk of intracranial extension, including conditions like cavernous sinus thrombosis, meningitis, subdural empyema, epidural abscess, subarachnoid inflammation, or involvement of the brain parenchyma [2]. It is often associated with a prior sinus surgery or a direct trauma to the frontal bone, as it happened in our case [3]. PPT presents predominately during adolescence, a period when the frontal sinuses are developing and experiencing an increased vascular growth with larger diploic veins [4]. Symptoms of PPT include headache, periorbital swelling, fever, purulent rhinorrhea, vomiting, and signs indicating meningitis or encephalitis [5]. If the inferior wall of the frontal sinus is affected, the infection may extend to the orbits, potentially resulting in conditions such as orbital cellulitis, an intraorbital abscess, preseptal cellulitis, or orbital phlegmon [6]. Approximately 40 percent of PPT cases present with an intracranial component, which is associated with a higher risk of mortality and morbidity [7]. Tsai et al. documented that all six paediatric patients with the Pott puffy tumour in their study experienced intracranial complications [8]. A CT scan is highly effective as an initial imaging method due to its speed, widespread availability, and ability to provide detailed images of the bone structures [9]. MRI is more effective in detailing intracranial pathology, dural sinus thrombosis and bone oedema associated with PPT. Bone scintigraphy using Technetium-99m Methylene Diphosphonate (Tc-99m MDP) might detect early osteomyelitis more effectively than CT scans, although its sensitivity is limited when acute sinusitis is present [10]. In circumstances where advanced imaging tools are not available, an X-ray can also help us in diagnosing the disease with a high index of clinical suspicion. The microorganisms frequently identified in cultures from Pott’s puffy tumour include Staphylococcus aureus, Streptococcus species, and anaerobic bacteria. Occasionally, Proteus, Fusobacterium, Bacteroides, and Pseudomonas may also be involved, although less commonly [11]. In our case, the culture grew MRSA, for which extended antibiotics had to be given to the patient as the proper antibiotic treatment is indispensable for treating PPT according to the result of bacteriological analysis. Other treatments of PPT and its intracranial complications involve the use of nasal decongestants, sinus washouts, percutaneous aspiration, trephination, endoscopic sinus surgery, and craniotomy [12].

## Limitations

A limitation of our study is that we have presented a single case report of PPT. A multi-centric study with a larger cohort could throw more insights into the disease pattern, presentations and complications.

## Conclusion

PPT, although uncommon in this era of antibiotics, can still be encountered. It requires immediate treatment with broad-spectrum intravenous antibiotics for 4 to 6 weeks along with surgical interventions depending on the clinical and radiological findings. A timely diagnosis and the appropriate treatment by a multidisciplinary team are crucial in reducing the morbidity and mortality linked to this rare condition, especially in cases with intracranial extension.

## Data Availability

This is a case report. No new data were created or analysed in this study. Data sharing is not applicable to this article.

## Financial assistance

No financial assistance has been provided for this research.
